# Response to “On the impact of incomplete taxon sampling on the relative timing of gene transfer events”

**DOI:** 10.1371/journal.pbio.3002557

**Published:** 2024-03-19

**Authors:** Théo Tricou, Eric Tannier, Damien M. de Vienne

**Affiliations:** 1 Université Clermont Auvergne, INRAE, VetAgro Sup, UMR EPIA, F-63122 Saint-Genès-Champanelle, France; 2 Univ Lyon, Université Lyon 1, CNRS, Laboratoire de Biométrie et Biologie Évolutive UMR5558, F-69622 Villeurbanne, France; 3 INRIA Grenoble Rhône-Alpes, F-38334 Montbonnot, France

In a previous article [1], we used simulations to show how acknowledging the existence of extinct and unsampled lineages, or “ghosts,” could substantially influence conclusions regarding gene flow derived from gene tree branch lengths analyses. We mentioned that this could in particular challenge the claim by Pittis and Gabaldon [2,3] that genes of alpha-proteobacterial origin are more recently transferred than those originating from actinobacteria.

Bernabeu and colleagues [4] propose a critique of our results based on 2 additional interesting analyses.

The first one, summarized in their Fig 1C, is a description of the conditions under which the conclusions of Pittis and Gabaldon [2,3] can actually be reversed (“conditions to shift” in their terminology), thus acknowledging this possibility. Bernabeu and colleagues conclude that these conditions are “rather restrictive” or “highly constrained,” suggesting that they are unlikely. This calls for 3 comments. One is that the list of conditions they give can be slightly relaxed. Indeed, it is not necessary that the earlier transfer (from alpha-proteobacteria) originates from a ghost lineage (as in Fig 1C in [4]). The shift is also obtained if this transfer comes directly from a non-ghost alpha-proteobacteria (α1 in their Fig 1) at an earlier time than the transfer from a ghost actinobacteria. This decreases the restrictivity of the conditions to shift. Another comment is that the conditions are not necessarily independent. Constraining the events to happen in a limited timeframe (the FECA-to-LECA period) is presented as a condition that decreases the chance that it occurs at all [4], but according to our analyses (Fig 6B in [1]) it may at the same time increase the probability that it leads to a shift. A last comment is that “rather restrictive” and “highly constrained” are vague and subjective qualifications. An opposite intuition is arguable: if we consider that most of the microbial diversity is unknown [5], and if we observe that numerous complete clades (such as the CPR, which represents a large part of the ToL presented as Fig 1A in Ref [4]) are probably missing from such an analysis, the conditions for shift seem sufficiently plausible to question the robustness of the stem-length based claims. Further work is needed to transform these intuitions into a probability and assess the likelihood that the conclusions of Pittis and Gabaldon [2,3] can be reversed (or not) when acknowledging the overwhelming presence of ghosts. Our simulations [1] were an attempt to quantify this effect. Surely they are not sufficient to give a definitive answer, because they were indeed not informed by all the knowledge we can have about the tree of life. At least, and in the light of the work of Bernabeu and colleagues [4], the question remains, in our view, largely open.

The second analysis of Bernabeu and colleagues [4] explores the sensitivity of the probability to reverse the conclusions of Pittis and Gabaldon [2,3] to the extinction rate. Using new simulations, they show that when 1% of the total number of species are sampled, increasing this rate to 0.9 reduces the proportion of incorrect predictions to 32%, compared with approximately 40% and 50% for rates of 0.5 and 0, respectively (Fig S3 in ref [4]). This sensitivity analysis is interesting, as it usefully complements our simulations (for which we recognize the noted error in the report of the simulation parameters in the text). It confirms that many combinations of parameters can influence the probability of shifts in one direction or the other. This is convergent with our claims that high uncertainties are associated with results based on the ghost-unaware stem-length method, even the smallest probability of shift equal to 0.32.

Finally, Bernabeu and colleagues [4] rightly recall that the possible effect of ghosts has been recognised before. Indeed, Hahn and colleagues [6] call for a “cautious” interpretation of the results; Susko and colleagues [7] recognise a possible “caveat”; Pittis and Gabaldon [2] test the possible effect of ghosts (in Section 4 of their supplementary material) by uniformly sampling alpha-proteobacteria and find no effect on the results. We hope that our work [1], along with the response by Bernabeu and colleagues [6], and a growing body of other studies ([8–10], to only cite a few), will help ghosts to gain a place in scientific articles that better reflects their thought importance in nature; that ghosts will not be confined to “supplementary data,” “additional remarks,” and quick control experiments anymore, but will find their place in main texts, figures, and hypotheses of future research.
